# Mechanistic insight into internal conversion process within Q-bands of chlorophyll *a*

**DOI:** 10.1038/s41598-017-11621-2

**Published:** 2017-09-12

**Authors:** Elena Meneghin, Cristina Leonardo, Andrea Volpato, Luca Bolzonello, Elisabetta Collini

**Affiliations:** Department of Chemical Sciences, University of Padova, Padova, Italy

## Abstract

The non-radiative relaxation of the excitation energy from higher energy states to the lowest energy state in chlorophylls is a crucial preliminary step for the process of photosynthesis. Despite the continuous theoretical and experimental efforts to clarify the ultrafast dynamics of this process, it still represents the object of an intense investigation because the ultrafast timescale and the congestion of the involved states makes its characterization particularly challenging. Here we exploit 2D electronic spectroscopy and recently developed data analysis tools to provide more detailed insights into the mechanism of internal conversion within the Q-bands of chlorophyll *a*. The measurements confirmed the timescale of the overall internal conversion rate (170 fs) and captured the presence of a previously unidentified ultrafast (40 fs) intermediate step, involving vibronic levels of the lowest excited state.

## Introduction

Chlorophyll *a* (chl*a*) is the most abundant photosynthetic light harvester, widespread in cyanobacteria, green algae and terrestrial plants^1–3^. Chl*a* appears to be ubiquitous in oxygenic photosynthesis and its role is determined by its unique photochemical properties. For this reason, this pigment has been widely studied, not only to assess its role in biological functions, but also with the aim of developing molecular models for bio-inspired artificial systems^4–9^.

More recently, particular attention has been devoted to the ultrafast relaxation dynamics of chl*a*, in the context of its possible involvement in quantum mechanisms of energy and charge transport in biological complexes^9–19^. In these photosynthetic proteins, indeed, the characteristic signatures resulting from the peculiar intra-chromophore vibrational and electronic structure can overlap with the most interesting collective behavior resulting from exciton interaction. A crucial aspect consists, thus, in the preliminary characterization of the distinctive signatures of the isolated harvester pigments. 2D electronic spectroscopy (2DES) is particularly suited to this aim, thanks to the high degree of information contents of 2DES responses^20, 21^. Indeed, 2DES has already been employed for the characterization of the femtosecond dynamics of the first excited state of chl*a* to identify the vibrational modes more strongly coupled to the electronic transition, to distinguish the frequency of ground state and excited state modes, and to unveil the role of solvent and spectral diffusion^22–25^.

In this work the attention is mainly focused on the relaxation and decoherence dynamics within the so called Q-bands^26^, a crucial preliminary step for other ensuing processes, including the energy transfer and the primary charge separation in the reaction center. Several efforts have been paid to characterize the dynamics of this process and the complex mechanisms regulating it, but its ultrafast timescale makes this task particularly challenging^27–30^. Here we exploit 2DES datasets recorded using different laser bandwidths, combined with theoretical simulations, to provide more detailed insights into the internal conversion process and unravel the femtosecond dynamics of the downward excitation energy pathways.

The Gouterman’s four orbitals model^26^ predicts that the Q-bands of chl*a* spectrum are the result of two (possibly overlapping) independent electronic transitions called Q_*x*_ (S_0_ → S_2_) and Q_*y*_ (S_0_ → S_1_), with *x* and *y* indicating the polarization directions within the macrocycle plane. The bands related to those transitions are broadened by inhomogeneous effects and by the activation of low-frequency molecular vibrations. Higher energy vibronic transitions appear instead as separated sidebands, usually identified as Q_*y*_(0, 1) and Q_*x*_(0, 1), respectively. While the lower energy band at 15150 cm^−1^ is assigned to Q_*y*_(0, 0) transition, the assignments of the other transitions is not straightforward. Historically, two different interpretations of the Q-bands were suggested. The first, proposed in the ‘60s’^31^, identifies the Q_*x*_ component with the signal at 17400 cm^−1^, while the second, from the ‘80s’^32^, assigns that transition to the feature at 17000 cm^−1^.

More recent interpretations state that Q_*x*_ and Q_*y*_ transitions, far from being independent transitions, are strongly mixed. Since Q_*x*_ is nearly resonant with the vibronically active mode of Q_*y*_ at 1500 cm^−1^, the *x*-polarized intensity is split nearly equally into two components and thus it is distributed over the entire Q-band system^33–35^.

More hints about the nature of the states can be obtained through the analysis of the 2DES maps, where different electronic transitions can be distinguished and mapped at different positions in the 2D plots. Two sets of measurements have been performed using two different exciting bandwidths. In the first set, the exciting laser spectrum was tuned to cover mainly the S_0_ → S_1,0_ (Q_*y*_(0, 0)) transition to characterize the relaxation dynamics of the lowest excited state. In the second set, the exciting laser spectrum was moved towards higher energies to capture the relaxation dynamics between S_2_ and S_1_ states. It is now known that the particular choice of the exciting band has dramatic consequences on the shape of the 2DES spectra and on the amplitude distribution of the signal relative to vibrational coherences, because the finite bandwidth acts like a spectral filter with non trivial effects^36, 37^. The interpretation of the experimental data has therefore been supported by theoretical simulation of the 2DES responses, accounting for the effective spectral shape and time convolution of the exciting pulses^38^.

## Results and Discussion

**Figure 1:**
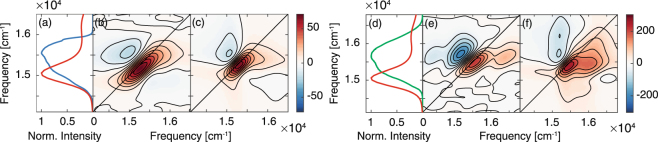
Comparison between experimental and simulated results in the two sets of measurements. Absorption spectrum of chl*a* in the Q-band region and spectral profile of the exciting pulses used in the first set of measurements in resonance with the *S*_0_ → S_1_ transition (**a**), and in the the second set of measures where the exciting pulse covers both S_1_ and S_2_ states (**d**). Experimental (**b**,**e**) and simulated (**c,f**) 2DES maps at population time *t*_2_ = 200 fs in the first and in the second set, respectively.

The results obtained in the first set of measurements (Fig. 1a–c and Supplementary Fig. S2) confirm the dynamic evolution of the Q_*y*_(0, 0) band already reported in the literature^22, 23^. The beating analysis highlighted, in particular, the contribution of three frequencies of 260, 420 and 745 cm^−1^ (Supplementary Fig. S4), corresponding to the vibrational modes more strongly coupled with the S_0_ → S_1_ transition, in agreement with previous measurements^22, 23^ and with the resonant Raman analysis^39^. The 2DES response, including the amplitude distribution of beatings at different frequencies, was simulated modelling the system with two electronic states (S_0_ and S_1_). The nuclear degrees of freedom were incorporated into the lineshape function associated to the electronic transition^40^. Frequencies and reorganization energies of the coupled vibrational modes were obtained from hole-burning experiments of chl*a* in ether^35^. Following standard nonlinear response function theory, the lineshape function was used as input to calculate the nonlinear response functions, linear absorption spectrum and 2DES spectra (see Supplementary Fig. S1).

The shape of the signals and the amplitude distribution of the main beating modes in the experimental 2DES maps reveal intensity patterns in full agreement with the two-level simulations (Fig. 1c), confirming that the 2DES response in this spectral region can be fully justified considering only the lower energy excited state S_1_.

More interesting results come from the second set of measurements. The evolution of the 2DES map in time reveals two different diagonal features at early times (Supplementary Fig. S5): a signal centered at 15500 cm^−1^ that corresponds to the blue tail of the S_1_ state, whose dynamics has been investigated in the previous set, and a second diagonal contribution appearing around 16360 cm^−1^. This peak is characterized by an ultrafast dynamics with amplitude halving in about 120 fs. In addition, 2DES maps are characterized by the presence of a strong lower diagonal cross peak between the recognized diagonal contributions, featuring a lively dynamics in the first hundred of femtoseconds after photoexcitation.

Differently from the previous set of data, in this case, the experimental behavior could not be fully justified by simulations including only the S_0_ and S_1_ states (Fig. 1e,f), suggesting that the higher energy diagonal signal cannot be interpreted as a vibronic state of S_1_. In agreement with the band attribution reported in refs 32, 33, 35, this signal has then be attributed to a second excited state, S_2_. The ultrafast dynamics moving the signal density from the higher to the lower diagonal cross peak and giving rise to the oscillating cross peak feature is thus depicting the S_2_ → S_1_ internal conversion. Below we report the analysis of the 2DES spectra that supports this attribution.

The attention has been focused first on the non oscillatory decay dynamics. The time evolution of the signals has been analyzed using a recently proposed methodology based on a global fitting of the whole 2DES dataset with complex multi-exponential functions^41^.

Briefly, the decay of the total complex signal at each point of the 2D map is fitted with a global function written as sum of N complex exponentials capturing simultaneously the population decay contributions and the oscillating components associated to coherent dynamics. The corresponding amplitudes plotted in a 2D map as a function of emission and excitation frequency build the so called 2D-DAS (decay associated spectra) and 2D-CAS (coherence associated spectra), respectively. This methodology provides a remarkably higher reliability in the identification of ultrafast decays and quickly damped oscillations with respect to other procedures since it fits simultaneously oscillating and non-oscillating components^41^. Moreover, this method allows retrieving at the same time the frequencies, damping times and amplitude maps for all the fitted components considering simultaneously real and imaginary parts (i.e. the full complex dataset). In order to minimize the possible contamination of coherent artifacts at early times, the fitting procedure has been applied to the data after exclusion of the first 30 fs.

**Figure 2:**
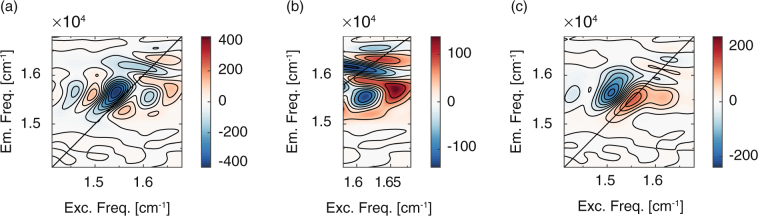
2D decay associated spectra (2D-DAS) from global fitting analysis illustrating the S_2_ → S_1_ population relaxation dynamics. (**a**) 2D-DAS associated to the 170 fs time constant. (**b**) Zoom of the previous map in a region corresponding to excitation energy of 16360 cm^−1^. (**c**) 2D-DAS associated to the 2 ps decaying component.

The analysis revealed that non oscillating dynamics is dominated by two time components, with time constants 170 fs and 2 ps, respectively. The corresponding 2D-DAS are reported in Fig. 2.

The 2D-DAS associated to the faster time constant reveals a very complex pattern. When the dynamics of a single transition of an isolated chromophore is investigated, time constants of hundreds of femtoseconds are typically associated to spectral diffusion processes, characterized by 2D-DAS with a well-defined signal amplitude distribution^41^, accounting for the rounding of the peak as the population time increases^23^. This is what has been recorded in the first set of measures (Supplementary Fig. S3(a)). The 2D-DAS of Fig. 2a presents a completely different behavior, suggesting a more complex dynamic process, likely involving more than one excited state. Negative (positive) features in 2D-DAS appear in regions of the 2D plots where the signal is rising (decaying) with the associated time constant. The negative signals in Fig. 2a can be justified invoking a rising population in a lower energy state, located where S_1_ is expected to contribute, as verified in the first measure set. The higher energy portion of the diagonal reveals instead a weak positive signal which is associated to a decaying feature that is located at energy compatible with the S_2_ state. We can therefore attribute this ultrafast time component to the relaxation of the population from S_2_ to S_1_ within the Q-bands.

A closer look to the 2D-DAS reveals however a more complicated distribution, especially in the spectral region corresponding to an excitation frequency of 16360 cm^−1^ (Fig. 2b): the signal amplitude along this coordinate shows, indeed, several sign changes. This evidence suggests the presence of complex relaxation dynamics between S_2_ and S_1_ states, involving several processes and intermediate states. Thus, the relaxation process is not characterized by a unique decaying component, but, more likely, by a distribution of components with possibly different time constants. In agreement with previous theoretical and experimental findings^30, 35^, the effective time constant for the overall process is found to be 170 fs.

**Figure 3:**
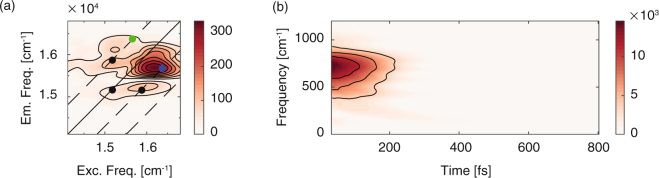
Analysis of the coherence dynamics of the second set of measures. (**a**) Fourier map at *ω*_2_ = 700 cm^−1^. Black dots pinpoint the positions where vibrational contributions of both S_0_ and S_1_ states are expected. Green and blue dots indicate the coordinates where the S_2_-S_1,*n*_ superposition should contribute. (**b**) Time-frequency map of the oscillations at coordinates (16360, 15660) cm^−1^, highlighting the strong contribution of an quickly damped oscillating signal centered at about 700 cm^−1^.

More details can be extracted from the oscillatory dynamics. After removing the non-oscillatory decaying part of the signal, the resulting oscillating residues have been Fourier transformed to generate the so-called Fourier maps^15, 42–46^. Not surprisingly, we could verify, also in this case, the presence of the vibrational frequencies already emerged in the first set and attributed to vibrational coherences of S_0_ and S_1_ states. The overall beating behavior of the 2DES signal in this spectral region is however dominated by a quickly damped oscillating signal centered at about 700 cm^−1^, whose Fourier map is shown in Fig. 3a, and contributing predominantly at coordinates (16360, 15660) cm^−1^ (blue dot in Fig. 3a) and, to a lower extent, on the symmetric upper diagonal position (green dot in Fig. 3a).

The dephasing dynamics of this oscillating signal has been characterized through two different approaches recently developed^41, 47^. First, the same global fitting method employed to characterize the non-oscillating dynamics has been applied. The results confirm that the main oscillating component is a damped coherence with frequency centered at about 700 cm^−1^ and dephasing time of 40 fs (see also Supplementary Fig. S6).

A more direct visualization of the beating dynamics can be obtained studying the oscillating residuals with a time-frequency transform (TFT) method^47^. Figure 3b reports the time-frequency map obtained analyzing the oscillating trace at (16360, 15660) cm^−1^ position (blue dot in Fig. 3a). In agreement with the results of the global fitting, the plot clearly shows a strong component with frequency centered at about 700 cm^−1^, bandwidth of about 200 cm^−1^ and dephasing time in the order of 50 fs. The quickly damped behavior explains the high indeterminacy in the frequency domain and thus the broadening of the corresponding Fourier spectrum.

**Figure 4:**
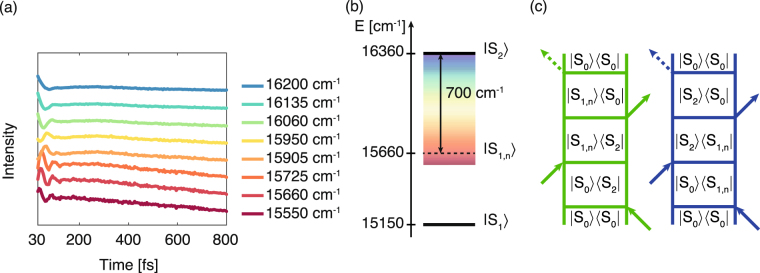
S_2_-S_1,*n*_ decoherence process. (**a**) Evolution of 2DES signal in *t*_2_ for traces extracted at different positions along a vertical line with excitation energy equal to 16360 cm^−1^. The values of the emission frequency coordinate for the different traces are reported in the legend. See also Supplementary Fig. S7. (**b**) Energy level diagram illustrating the main states involved in the internal conversion process as deduced from the dynamics of the 2DES response. (**c**) Feynman diagrams accounting for the oscillating contributions at the cross peak positions (pinpointed in Fig. 3a with the same color code).

The position and the dynamic behavior of these signals can be explained invoking relaxation pathways that involve coherences between S_2_ state and vibronic states of the first excited state, S_1,*n*_, as illustrated in Fig. 4c. Figure 4a supports this interpretation: at different positions along a vertical line at excitation energy of 16360 cm^−1^, the 2DES signal revealed that the central frequency of the above-mentioned damped component progressively increases moving away from the diagonal towards lower emission energies. This implies that, while the central frequency of the coherent signal roughly corresponds to a superposition between S_2_ and a vibronic state at about 510 cm^−1^ above the vibrational ground state of S_1_, the full manifold of vibrational levels is involved, as sketched by the color scale in Fig. 4a,b.

On the other hand, also in agreement with previous 2DES measurements^22^, no signatures of a S_2_-S_1,0_ coherent dynamics have been found. This seems to indicate that, overall, the internal conversion leading to the relaxation of the excitation at the bottom of the Q-band (S_1,0_) is an incoherent process with a timescale of about 170 fs. Nevertheless, the vibronic states of S_1_ are strongly involved as intermediates in the relaxation process. The timescale of these intermediate steps cannot be fully captured looking directly at the population dynamics (Fig. 2) but an upper limit for the associated time constants can be estimated considering the dephasing time of the S_2_-S_1,*n*_ superpositions prepared by the laser excitation, in the order of 40 fs. Given the fast timescale involved, this time constant could be affected by a large uncertainty. However, the crucial point of this analysis is the experimental detection of a quickly decaying signature of the S_2_-S_1_ mixing, possibly justifying the fast interconversion between the two states. It is interesting to note that the quick Q_*x*_ (S_2_) decoherence captured by our data was already theoretically predicted and justified in terms of Q_*x*_-Q_*y*_ mixing^35^.

The following internal conversion mechanism can thus be proposed: following excitation on the high energy side of the Q-bands, the excitation energy is quickly (about 40 fs) redistributed among vibronic states of the S_1_ level before being eventually directed to the bottom of the band in 170 fs.

## Conclusions

In conclusion, the 2DES characterization of chl*a* discussed in this work gives a new and detailed mechanistic insight on the relaxation and dephasing dynamics of internal conversion process within the Q-bands. The analysis of the dynamics response by means of recently developed methodologies confirmed the timescale of the overall internal conversion rate (170 fs) and captured the presence of an intermediate step involving vibronic states S_1,*n*_, in particular a state at 510 cm^−1^ above the vibrational ground state of electronic state S_1_. The presence of such intermediate process, suggested by the analysis of the population dynamics, could be better characterized studying the evolution of the S_2_-S_1,*n*_ coherences prepared by the laser excitation. The investigation on their dephasing dynamics provided indeed an estimate of the time constant associated with this intermediate step (40 fs). While the involvement of vibronic levels of S_1_ and the overall ultrafast timescale of the relaxation process are well established facts^30^, this work represents a step ahead of the current knowledge because not only it demonstrates that vibronic states indeed contribute, but also it provides a direct quantitative proof of how they affect the process and in which timescale.

The characterization of these mechanistic details represents an important piece of information in the wider context of the photosynthetic energy transport in chl*a*-based antenna protein complexes. Non radiative relaxation of high-energy excited states to the lowest excited state represents indeed the first stage of photosynthesis. The ultrafast timescale of this relaxation and the congestion of energy levels within the Q-band region make the full characterization of this process particularly challenging, as demonstrated by the continuous experimental and theoretical attempts appearing in the literature. We therefore expect these findings to be particularly important in the future interpretation of 2DES coherent response of biological complexes bearing chl*a*.

## Methods

### Sample preparation and characterization

Chl*a* from spinach was purchased from Sigma Aldrich and used without further purification. The sample solutions were prepared dissolving chl*a* in MeOH with a concentration of about 50 *μ*M, leading to an optical density of about 0.3 on the maximum of the Q-bands with a pathlength of 1 mm. In these conditions chl*a* is not expected to form aggregates and, indeed, we verified that the normalized steady-state absorption spectrum do not show any significant modification lowering the concentration down to two orders of magnitude. Steady-state absorption spectra were acquired before and after each scan to control that no degradation of the sample occurred during the 2DES measurements.

### 2D electronic spectroscopy

2DES measurements have been performed with the setup described in ref. 48. Briefly, the output of a 800 nm, 3 KHz Ti:Sapphire laser system (Coherent Libra) is converted in a visible broad pulse in a non-collinear optical amplifier (Light Conversion TOPAS White). The transform-limited condition for the pulses at the sample position is achieved through a prism compressor coupled with a Dazzler pulse shaper for the fine adjustment. The pulse duration is optimized through FROG measures. The pulses energy at the sample position is reduced until 7 nJ per pulse by a broadband half-waveplate/polarizer system. The 2DES experiment relies on the passively phase stabilized setup. The laser output is splitted into four identical phase-stable beams (three exciting beams and a fourth beam further attenuated of about 3 orders of magnitude and used as Local Oscillator, LO) in a BOXCARS geometry using a suitably designed 2D grating. Time delays between pulses are modulated by pairs of 2° CaF_2_ wedges. One wedge of each pair is mounted onto a translation stage that regulates the thickness of medium crossed by the exciting beam and provides a temporal resolution of 0.07 fs. 2DES experiments were performed tuning the TOPAS White in two different spectral ranges as shown in Fig. 1a,d. The pulse duration and the spectral bandwidth (FWHM) are 24 fs and 610 cm^−1^ in the first set of measures; 18 fs and 820 cm^−1^ in the second set. Rephasing spectra were acquired for population times ranging from 0 to 800 fs in 5 fs increments, with each experiment repeated three times to ensure reproducibility.

### Data Availability

The datasets generated and analyzed during the current study are available from the corresponding author on reasonable request.

## Electronic supplementary material

Supplementary information
