# Supplementary material for: Mechanistic insight into internal conversion process within Q-bands of chlorophyll a
Source: Sci Rep. 2017 Sep 12;7:11389. doi: 10.1038/s41598-017-11621-2 (PMC5595816; doi:10.1038/s41598-017-11621-2)
Supplement: Supplementary file 1 — Supplementary information [file 41598_2017_11621_MOESM1_ESM.pdf]

Supplementary Information
Mechanistic insight into internal conversion process within Q-bands of chlorophyll *a*

Elena Meneghin[a], Cristina Leonardo[a], Andrea Volpato[a], Luca Bolzonello[a] and Elisabetta Collini*[a]

[a] Department of Chemical Sciences, University of Padova, Padova
* corresponding author e-mail: elisabetta.collini@unipd.it

1 2DES spectra calculation

2DES spectra are calculated using a framework of methods already presented in several references, see for example [1, 2]. The calculation is based on third order perturbation theory [3, 4], where a response function is fully convoluted with all the exciting pulses [5], and the spectral density is described using the Brownian oscillator model [6]. The system is modeled as a two level system, ground state (S_0) and excited state (S_1), with vertical transition energy $\omega_{S_0 \to S_1} \equiv \omega_0 = 15400\ \text{cm}^{-1}$.

Within the perturbation theory framework, the third order nonlinear optical response is given by the third order polarization $P^{(3)}(t)$. It is calculated as the convolution of three excitation electric fields $E(\mathbf{r}, t)$ with the third order response function $S^{(3)}(t_1, t_2, t_3)$ as

$$\begin{aligned} P^{(3)}(t) = &\int_0^\infty dt_3 \int_0^\infty dt_2 \int_0^\infty dt_1 S^{(3)}(t_1, t_2, t_3) \\ &\times E(\mathbf{r}, t - t_3) E(\mathbf{r}, t - t_2 - t_3)(\mathbf{r}, t - t_1 - t_2 - t_3) \end{aligned} \tag{1}$$

where t_1, t_2 and t_3 are the times of each field interaction and $\mathbf{r}$ is the propagation direction of the fields.

The response function contains all the information accessible through non-linear spectroscopy. For a two level system only four contributions sum up to get the full response, two for the rephasing response: $S^{(3)}_{\text{reph}} = R_2 + R_3$; and two for the non-rephasing response: $S^{(3)}_{\text{non-reph}} = R_1 + R_4$. The non-linear response

function is calculated using the following expressions

$$
\begin{aligned}
R_1 &= |\mu|^4 \exp\{-\omega_0 t_1 - \omega_0 t_3 - g(t_1) - g^*(t_2) - g^*(t_3) + g(t_1 + t_2) \\
&\quad + g^*(t_2 + t_3) - g(t_1 + t_2 + t_3)\} \\
R_2 &= |\mu|^4 \exp\{+\omega_0 t_1 - \omega_0 t_3 - g^*(t_1) + g^*(t_2) - g(t_3) - g^*(t_1 + t_2) \\
&\quad - g^*(t_2 + t_3) + g^*(t_1 + t_2 + t_3)\} \\
R_3 &= |\mu|^4 \exp\{+\omega_0 t_1 - \omega_0 t_3 - g^*(t_1) + g(t_2) - g^*(t_3) - g^*(t_1 + t_2) \\
&\quad - g(t_2 + t_3) + g^*(t_1 + t_2 + t_3)\} \\
R_4 &= |\mu|^4 \exp\{-\omega_0 t_1 - \omega_0 t_3 - g(t_1) - g^*(t_2) - g(t_3) + g(t_1 + t_2) \\
&\quad + g(t_2 + t_3) - g(t_1 + t_2 + t_3)\},
\end{aligned}
\tag{2}
$$

where μ is the transition dipole moment, and $g(t)$ is the lineshape function which accounts for the spectral broadening of the signal. The function $g(t)$ is an integral transformation of the spectral density $C(\omega)$ of the bath fluctuations coupled to the electronic transition, and it is specified as

$$
\begin{aligned}
g(t) =& \frac{1}{2\pi} \int_{-\infty}^{\infty} d\omega \, [1 - cos(\omega t)] \coth\left(\frac{\hbar\omega}{2k_B T}\right) \frac{C(\omega)}{\omega^2} \\
&+ \frac{i}{2\pi} \int_{-\infty}^{\infty} d\omega \, [sin(\omega t) - \omega t] \, \frac{C(\omega)}{\omega^2},
\end{aligned}
\tag{3}
$$

where k_B is the Boltzmann constant and T is the absolute temperature.

Coupled molecular vibrations and interactions with the external bath on multiple time scales are modeled with the Brownian oscillator formalism [6] using under-damped and over-damped oscillators, respectively. The under-damped component of the spectral density, arising from molecular vibrations, is specified as

$$
C_{\text{under-damped}}(\omega) = \sum_i \frac{2\sqrt{2}\lambda_i \gamma_i \omega_i^2 \omega}{(\omega - \omega_i^2) + 2\gamma_i^2 \omega^2}, \tag{4}
$$

where λ_i is the reorganization energy, γ_i is the damping constant and ω_i is the frequency of the molecular vibration. Frequencies and reorganization energies of the coupled vibrational modes were obtained from hole-burning experiments of chl$a$ in ether [7]. Damping constants were set equal to 0.5 ps^{-1}. In order to obtain a good match with the experimental linear absorption and third order signal, two over-damped oscillators were employed, the corresponding spectral density is specified as

$$
C_{\text{over-damped}}(\omega) = \sum_{i=1}^{2} \frac{2\lambda_i \gamma_i \omega}{\omega^2 + \gamma_i^2}, \tag{5}
$$

where λ_i is the reorganization energy and γ_i is the inverse of the fluctuation correlation time. In particular $\lambda_1 = 125\text{cm}^{-1}$, $\lambda_2 = 25\text{cm}^{-1}$, $\gamma_1 = 0.25\text{ps}^{-1}$ and $\gamma_2 = 10^3\text{ps}^{-1}$.

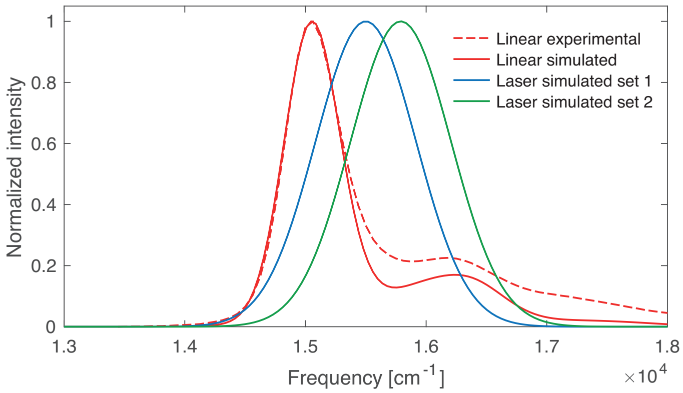

Figure S 1: Simulated (solid red line) and experimental (dashed red line) steady-state absorption spectra of chl$a$ in MeOH. Spectral profile of the exciting pulse used in the simulation of the first (blue) and second (green) set of measures.

Given the lineshape function, the linear absorption spectrum is promptly calculated as

$$ABS(\omega) = |\mu|^2 Re \left[\int_{-\infty}^{\infty} dt \exp\{i(\omega - \omega_0)t - g(t)\} \right]. \tag{6}$$

Rephasing and non-rephasing responses were convoluted with the pulse temporal profile, as in Eq. 1, following the procedure described in ref. [5]. Two different laser profiles were used to model experimental laser conditions. Simulated linear absorption spectra and simulated laser spectra are reported in Fig. S1.

2 Data fit procedure

The time constants, frequencies, amplitudes and dephasing times characterizing both the coherent and the non-coherent dynamics of the 2D maps are the results of a global fitting procedure based on the variable projection algorithm. Briefly, the decay of the total complex signal at each point of the 2D map is fitted with a global function written as sum of N complex exponentials, where the n-th component can be expressed as

$$c_{nk} = a_n e^{-t_k/\tau_n} e^{i\omega_n t_k} \tag{7}$$

where t_k indicates the k-th population time, a_n is the complex amplitude, τ_n is the decay constant and ω_n is the frequency.

Components with $\omega_n = 0$ describe population decay contributions, whereas components with $\omega_n \neq 0$ represent oscillating components associated to coherent

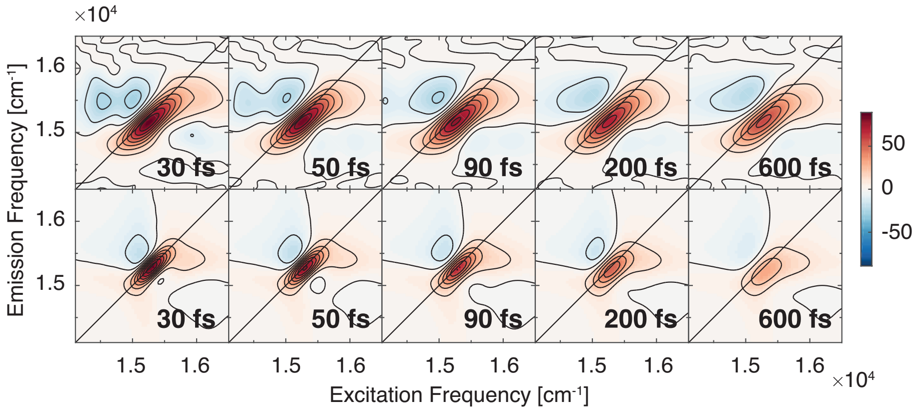

Figure S 2: Evolution of 2DES rephasing maps of chl$a$ in MeOH in the population time range 30-600 fs: experimental (first line) and simulated (second line) spectra.

dynamics along t_2. The corresponding amplitude a_n, plotted in a 2D map as a function of ω_1 and ω_3, builds the so called 2D-DAS (decay associated spectra) and 2D-CAS (coherence associated spectra). In order to minimize the possible contamination of coherent artifacts at early times, the fitting procedure has been applied to the data after exclusion of the first 30 fs.

3 Additional 2DES data

Two different sets of 2DES data have been recorded on methanol (MeOH) solutions of chlorophyll a (chl$a$). In the first set the exciting laser pulse was tuned to be resonant with the $S_0 \to S_1$ transition to confirm the dynamics of the lowest excited state (S_1). In the second set, the exciting laser spectrum was moved towards higher energies to capture the relaxation dynamics between S_2 and S_1 states.

3.1 First dataset

Fig. S2 reports the evolution of the experimental rephasing signal and the comparison with the simulated results. The good agreement confirms that in this spectral region the dynamics is dominated by S_1 relaxation.

The global fitting analysis [8] revealed two non-oscillating components: (i) a faster contribution (250 fs) mainly associated to spectral diffusion; (ii) a slower decay (3.3 ps) describing the relaxation of the whole map. The 2D-DAS related to the two processes are reported in Fig. S3.

The analysis of the coherent part of the 2DES signal revealed several components. The 2D-CAS of the main oscillating contributions at 260 cm^{-1}, 420

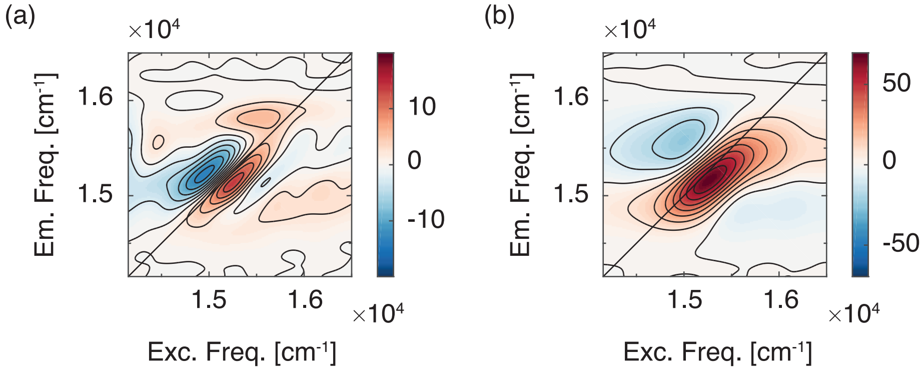

Figure S 3: 2D-DAS of the two non-oscillating components detected in the rephasing signal of the first set of measures associated to (a) 250 fs and to (b) 3.3 ps time constants.

cm^{-1}, 550 cm^{-1}, 745 cm^{-1} and 780 cm^{-1}, are reported in Fig. S4. Black dots pinpoint the positions where the characteristic chair-pattern of vibrational coherences should contribute, in agreement with ref. [6].

3.2 Second dataset

Fig. S5 (first line) shows the evolution of the 2DES maps along population time. At early times, two distinct diagonal features can be distinguished: (i) a signal elongated along the diagonal and centered at 15400 cm^{-1} that corresponds to the blue tail of the $S_0 \rightarrow S_1$ band; (ii) a second diagonal contribution appearing close to 16360 cm^{-1}. The position of this signal is compatible with the red part of the $S_0 \rightarrow S_2$ band. This peak is characterized by an ultrafast dynamics. In addition, 2DES maps are characterized by the presence of a strong cross-peak between the recognized diagonal contributions that suggests the presence of an ultrafast relaxation dynamics from S_2 to S_1.

Differently from the first set of data where an excellent agreement between experimental and theoretical spectra could be achieved, in the second set of measure the simulation could not fully capture all the features in the experimental maps. This suggest that indeed the relaxation of S_1 is not enough to describe the dynamics in this spectral region and that the presence of additional excited state is needed to justify the experimental response. First, the simulation does not capture the presence of the cross peak at coordinates (16360, 15660) cm^{-1} commented in the main text and attributed to the $S_2 \rightarrow S_1$ relaxation. Note that the simulation shows instead a cross peak at (16000, 15500) cm^{-1}, accounting for a vibronic sideband of the $S_0 \rightarrow S_1$ transition. The positions and the dynamic behavior of this feature is however completely different. This suggest that indeed the cross peak at (16360, 15660) cm^{-1} cannot be justified accounting only for the vibronic properties of the S_1 state. Second, the experimental

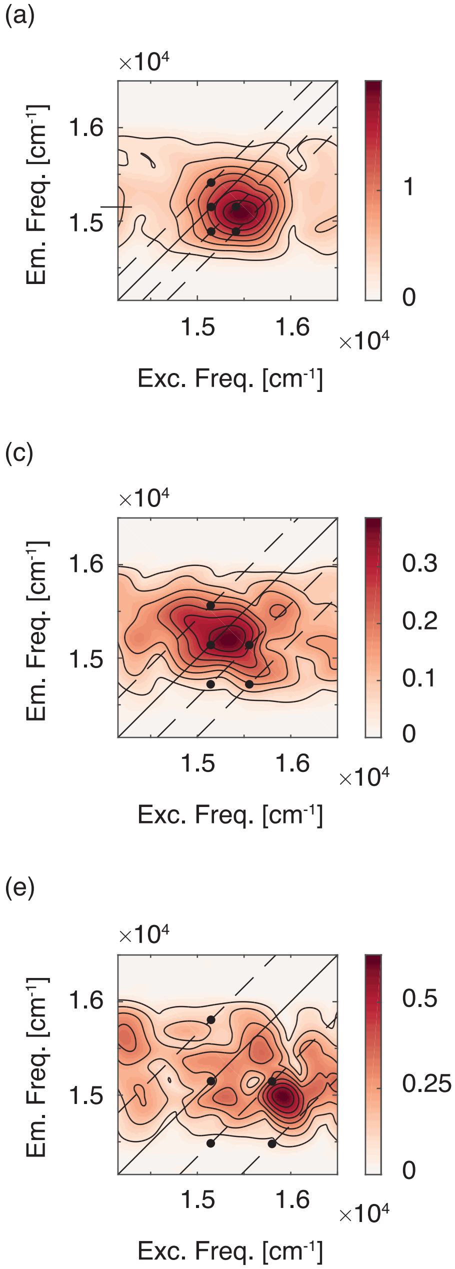

Figure S 4: 2D-CAS of the oscillating components detected in the first set of measures. (a) $\omega_2 = 260$ cm^{-1}, $t_{deph} = 420$ fs; (b) $\omega_2 = 420$ cm^{-1}, $t_{deph} \gg 1$ ps; (c) $\omega_2 = 550$ cm^{-1}, $t_{deph} \gg 1$ ps; (d) $\omega_2 = 745$ cm^{-1}, $t_{deph} = 1$ ps; (e) $\omega_2 = 780$ cm^{-1}, $t_{deph} = 320$ fs.

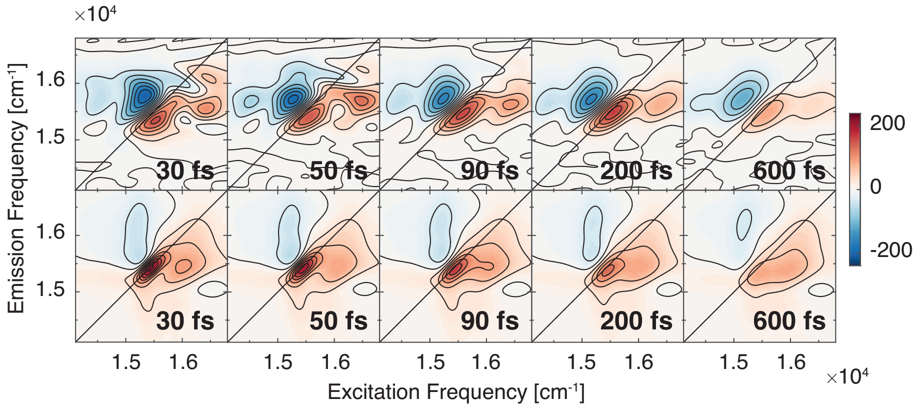

Figure S 5: Evolution of 2DES rephasing maps of chl$a$ in MeOH in the population time range 30-600 fs: experimental (first line) and simulated (second line) spectra.

data revealed a more intense negative signal above the diagonal that most likely arises from an excited state absorption (ESA) $S_1 \rightarrow S_n$.

The global fitting analysis [8] disentangled two non-oscillating components (170 fs and 2 ps) and the related 2D-DAS have been already reported in the main text in Fig. 2.

More interesting behaviors have been caught in the analysis of the oscillating part of the 2DES signal. The global fit, indeed, captured a quickly damped (40 fs) beating with average frequency of 700 cm^{-1}. 2D-CAS obtained for this component are reported in Fig. S6. Note that the total 2D-CAS of Fig. S6(c) shows a very good agreement with the Fourier map reported in Fig. 3(a) of the main text.

The strong oscillating behavior during the first hundred of femtoseconds and the decay of signal density from higher to lower energy states in the same timescale is furtherly highlighted in Fig. S7. The figure shows the evolution along the population time of a vertical slice of the signal at excitation frequency of 16360 cm^{-1}.

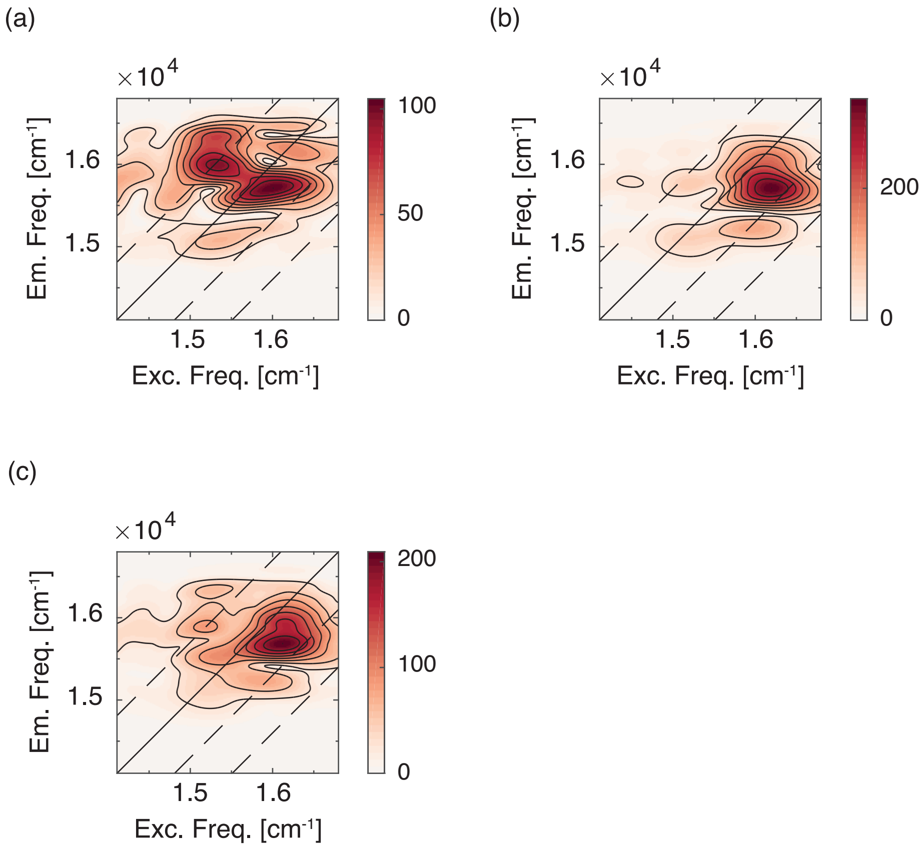

Figure S 6: 2D-CAS of the damped oscillating component with 700 cm^{-1} central frequency and 40 fs dephasing time. The components of the map at negative (-700 cm^{-1}) and positive (+700 cm^{-1}) frequencies are reported in panel (a) and (b), respectively. Panel (c) summarizes the total signal.

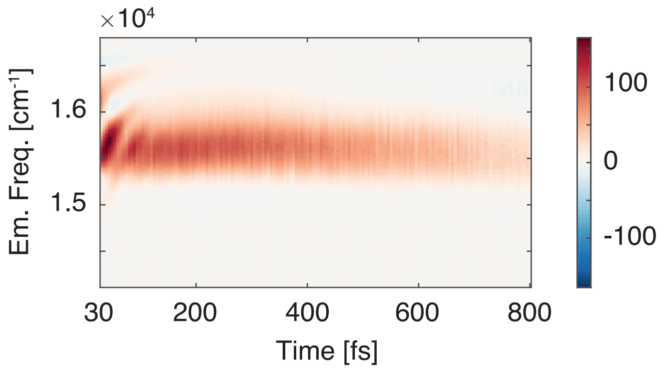

Figure S 7: Time evolution of a vertical slice of the signal extracted at 16360 cm^{-1} excitation frequency. The strongly oscillating behavior in the first hundred of femtoseconds can be easily recognized.

References

[1] N. Christensson, F. Milota, J. Hauer, J. Sperling, O. Bixner, A. Nemeth, and H. F. Kauffmann, "High frequency vibrational modulations in two-dimensional electronic spectra and their resemblance to electronic coherence signatures," *J. Phys. Chem. B*, vol. 115, no. 18, pp. 5383–5391, 2011.

[2] J. R. Caram, A. F. Fidler, and G. S. Engel, "Excited and ground state vibrational dynamics revealed by two-dimensional electronic spectroscopy," *J. Chem. Phys.*, vol. 137, no. 2, p. 024507, 2012.

[3] S. Mukamel, *Principles of nonlinear optical spectroscopy.* Oxford: Oxford University, 1995.

[4] M. Cho, *Two-dimensional optical spectroscopy.* Boca Raton: CRC Press, 2009.

[5] D. Abramavicius, V. Butkus, J. Bujokas, and L. Valkunas, "Manipulation of two-dimensional spectra of excitonically coupled molecules by narrow-bandwidth laser pulses," *Chem. Phys.*, vol. 372, no. 1-3, pp. 22–32, 2010.

[6] V. Butkus, D. Zigmantas, L. Valkunas, and D. Abramavicius, "Vibrational vs. electronic coherences in 2D spectrum of molecular systems," *Chem. Phys. Lett.*, vol. 545, pp. 40–43, 2012.

[7] J. R. Reimers, Z.-L. Cai, R. Kobayashi, M. Rätsep, A. Freiberg, and E. Krausz, "Assignment of the Q-bands of the chlorophylls: coherence loss via Qx - Qy mixing," *Sci. Rep.*, vol. 3, no. 1, p. 2761, 2013.

[8] A. Volpato, L. Bolzonello, E. Meneghin, and E. Collini, "Global analysis of coherence and population dynamics in 2D electronic spectroscopy," *Opt. Express*, vol. 24, no. 21, pp. 24773–24785, 2016.
